# Glatiramer acetate treatment effects on gene expression in monocytes of multiple sclerosis patients

**DOI:** 10.1186/1742-2094-10-126

**Published:** 2013-10-17

**Authors:** Madhan Thamilarasan, Michael Hecker, Robert Hermann Goertsches, Brigitte Katrin Paap, Ina Schröder, Dirk Koczan, Hans-Jürgen Thiesen, Uwe Klaus Zettl

**Affiliations:** 1 Institute of Immunology, University of Rostock, Schillingallee 68, Rostock 18057, Germany; 2 Department of Neurology, Division of Neuroimmunology, University of Rostock, Gehlsheimer Straße 20, Rostock 18147, Germany

**Keywords:** Glatiramer acetate, Relapsing-remitting multiple sclerosis, Monocytes, Gene expression profiling, Microarray analysis

## Abstract

**Background:**

Glatiramer acetate (GA) is a mixture of synthetic peptides used in the treatment of patients with relapsing-remitting multiple sclerosis (RRMS). The aim of this study was to investigate the effects of GA therapy on the gene expression of monocytes.

**Methods:**

Monocytes were isolated from the peripheral blood of eight RRMS patients. The blood was obtained longitudinally before the start of GA therapy as well as after one day, one week, one month and two months. Gene expression was measured at the mRNA level by microarrays.

**Results:**

More than 400 genes were identified as up-regulated or down-regulated in the course of therapy, and we analyzed their biological functions and regulatory interactions. Many of those genes are known to regulate lymphocyte activation and proliferation, but only a subset of genes was repeatedly differentially expressed at different time points during treatment.

**Conclusions:**

Overall, the observed gene regulatory effects of GA on monocytes were modest and not stable over time. However, our study revealed several genes that are worthy of investigation in future studies on the molecular mechanisms of GA therapy.

## Background

Multiple sclerosis (MS) is a chronic immune-mediated disease of the central nervous system (CNS). The auto-reactive behavior of the immune system in MS patients is associated with inflammatory lesions in the CNS and axonal demyelination. There is currently no cure for MS, but there are several therapies available as disease-modifying agents. The relapsing-remitting type of MS (RRMS) is mainly treated with immunomodulating drugs like interferon-beta (IFN-β) and glatiramer acetate (GA) [1,2].

GA (copolymer 1) is a first-line treatment option for RRMS. Different clinical trials have shown that GA treatment decreases the incidence of relapses and significantly reduces the number of gadolinium-enhancing lesions in magnetic resonance imaging (MRI) [3-6]. GA is not a defined chemical substance, but a standardized mixture of synthetic peptides. These peptides are made up of four different amino acids, glutamic acid, lysine, alanine and tyrosine (G-L-A-T), in a molar ratio of 1.5:3.6:4.6:1.0, assembled in a random order into polypeptide chains with a length of 40 to 100 residues [7]. This mixture of peptides was initially intended to mimic myelin basic protein (MBP) and to induce experimental autoimmune encephalomyelitis (EAE), the animal model of MS [8]. However, surprisingly, GA inhibited EAE in rodents and monkeys [9]. Today, GA has been well-established for MS therapy for more than a decade due to its beneficial clinical effects and its favorable safety profile.

According to pharmacokinetic studies, GA is quickly absorbed after subcutaneous administration, and it undergoes rapid degradation to amino acids and shorter peptides. Only 10% of the peptides remain at the site of injection after one hour [10]. A fraction of GA presumably enters the lymphatic circulation and reaches the regional lymph nodes where it modulates immune responses. Of note, patients treated with GA have the tendency to develop antibodies against it. However, the biological meaning of anti-GA antibodies remains controversial and it is unclear whether they may have a neutralizing or a beneficial effect in MS patients [11-14].

Different molecular mechanisms of action of GA have been proposed [7,15-18]. One postulated mechanism is that GA peptides act as altered peptide ligands (APL). An APL is a peptide, usually closely related to an agonist peptide in amino acid sequence, that induces a different function or partial response of T-cells specific for the agonist peptide as a result of the modified interaction with the T-cell receptor (TCR). During GA therapy, the partial activation of T-cells specific for MBP and other myelin antigens can induce peripheral tolerance, and this may contribute to the clinical effects of GA by preventing the attack of the myelin sheath around the nerves. Indeed, cross-reactivity of GA-specific T-cells with myelin antigens has been demonstrated, and there is evidence that prolonged exposure to GA results in anergy or depletion of GA-reactive cells that are possibly relevant in the pathogenesis of MS [19-23]. On the other hand, several studies have shown that GA induces a T helper cell type 1 (Th1) to Th2 shift in T-cells. Th1 and Th2 each produce a different combination of pro- and anti-inflammatory cytokines, respectively. The increase of GA-reactive Th2 cells during treatment is regarded as a central mechanism of action of GA [24-27]. These anti-inflammatory Th2-like cells were found to mediate regulatory functions as they can migrate into the brain and act suppressively at the sites of inflammation (local bystander suppression). This leads to a reduced activation and proliferation of auto-reactive immune cells, even if they recognize unrelated antigens and do not cross-react with GA [11].

Antigen-presenting cells (APC) are also believed to play a role in the immunomodulatory effects of GA therapy. The professional APC are dendritic cells and macrophages, which differentiate from circulating monocytes [28]. The interplay between APC and T-cells is fundamental in adaptive immune responses as well as in the pathophysiology of MS [29]. One suggested mechanism of GA is that it binds to major histocompatibility complex (MHC) molecules and thus competes with myelin antigens for their presentation on APC to T-cells. Specifically, GA can act as an antagonist of MBP/MHC at MBP-specific TCR and it is able to displace MBP from the binding site on MHC class II molecules [30,31]. On the other hand, GA was shown to change the properties of APC in such a way that they stimulate Th2-like responses. These APC are called type II APC. The effect of the drug on APC seems to depend on the cell type [11,32]. Weber *et al.* showed that GA inhibits monocyte reactivity and induces type II monocytes, which promote both Th2 differentiation and expansion of T regulatory cells (Treg). They observed that after GA administration in EAE, the pattern of cytokine production by monocytes switched towards an anti-inflammatory profile, characterized by down-regulation of pro-inflammatory cytokines (for example, IL12) and up-regulation of anti-inflammatory cytokines (for example, IL10) [33,34]. An increased IL10 production of monocytes has already been observed 72 hours post GA therapy initiation in a recent study by Ayers *et al.*[35]. Other studies confirmed that there is an increase in anti-inflammatory type II monocytes during GA therapy, and that the suppressor functions of these monocytes contribute to Th2 deviation of naive T-cells of MS patients [36-38]. Additionally, GA was shown to affect monocytes by increasing the expression of IL1RA while diminishing the production of IL1-β [39,40]. Recently, Caragnano *et al.* also observed a trend for IL1-β down-regulation in stimulated monocytes from GA-treated MS patients, and this was paralleled by lower levels of P2RX7, a receptor regulating cytokine production and apoptosis [41]. However, whether GA acts directly on monocytes *in vivo*, or whether the effects on monocytes are mediated by cytokines produced by GA-specific Th2 cells, is unclear as there is a dynamic feedback loop between human T-cell and APC responses [11,36].

However, GA not only modulates CD4+ T helper cell responses and binds to MHC class II molecules on APC. It has also been shown that GA incites an HLA class I-restricted, cytotoxic suppressor CD8+ T-cell response [42]. This may be mediated by heat shock proteins (HSPs) that bind extracellular antigens and mediate their cellular uptake. HSP-antigen complexes are then directed toward either the conventional class II pathway or the MHC class I pathway through cross-presentation [43,44]. In the similar way, GA peptides may bind to HSPs, and thus may be presented on MHC class I molecules resulting in an altered activation of T-cell subsets. This potentially leads to cytotoxic T-cells, which can kill CD4+ T-cells in a GA-specific manner [42]. In addition to its immunomodulatory effects, direct neuroprotective and even remyelinating properties have been ascribed to GA as well [45-49]. For instance, GA may foster repair after neurologic damage by stimulating the expression of neurotrophic factors like BDNF by various immune and CNS resident cells [50].

Over the last years, several research groups performed longitudinal gene expression profiling studies with microarrays to better understand the mechanisms of action of MS therapies. However, while the broad and rapid gene regulatory effects of IFN-β treatment in blood cells have been investigated extensively [51], there is only one such study for GA treatment: Achiron *et al.* measured the gene expression in peripheral blood mononuclear cells (PBMC) from 14 RRMS patients before and three months after initiation of GA therapy [52]. In their analysis, they identified 480 genes to be differentially expressed at the transcript level. They concluded that changes in the expression of immunomodulatory genes during GA therapy are important to reduce the activity of the disease [52].

The present study focuses on the effects of daily subcutaneous GA injections on the mRNA expression profile of monocytes in the peripheral blood. We were interested in monocytes because GA has been described as modulating these cells to promote Th2-like responses [11,31], but the *in vivo* effects have so far not been examined in a genome-wide and longitudinal manner. We obtained monocytes from RRMS patients immediately before as well as at four different time points after the start of GA therapy. The gene expression analysis was performed using microarrays. Genes that were found to be differentially expressed in response to GA therapy were then analyzed for biological functions and molecular interactions to derive new hypotheses on the molecular mechanisms of action of GA. This is the first study that investigates the transcriptome dynamics over the course of the therapy in a cell type-specific manner.

## Methods

### Blood sample collection

Eight Caucasian patients with diagnosed RRMS according to the revised McDonald criteria [53] were recruited for this study. The patients started a treatment with GA (Copaxone, Teva Pharmaceutical Industries Ltd., Petah Tikva, Israel) in 20 mg doses given daily as a subcutaneous injection. Five of the patients were not treated with any immunomodulatory or immunosuppressive drug prior to the onset of this study. Two patients (MS3 and MS5) received subcutaneous IFN-β, and one patient (MS8) received mitoxantrone (the last injection was four months ago) previously (Table 1). All patients were given routine care following the consensus treatment guidelines and recommendations of the German Society of Neurology (DGN). Blood samples were obtained from each patient at five different time points: before the first injection of GA (baseline) and after one day (that is, before the second injection) as well as after one week, one month and two months. Collection of blood was done by venipuncture with ethylenediaminetetraacetic acid (EDTA) as anticoagulant. An approximate volume of 15 ml whole blood was collected for each patient and each time point. In the clinical follow-up, the patients were assessed neurologically, monitored for relapses, and rated using the expanded disability status scale (EDSS) and cranial MRI. The study was approved by the ethics committee of the University of Rostock and carried out according to the Declaration of Helsinki. All patients gave written informed consent to be included in the study.

**Table 1:** Demographic data and clinical data of the eight patients

**Patient**	**Gender**	**Age at study onset**	**Disease duration**	**Previous treatment**	**EDSS at baseline**	**EDSS after 12 months**	**Relapses during first 12 months**	**cMRI at follow-up**
MS1	Female	41	0	None	1.5	1.5	0	-
MS2	Female	50	10	None	1.5	1.5	1	New lesion
MS3	Female	37	33	IFN-beta sc.	1.0	1.5	0	stable
MS4	Female	38	9	None	1.0	1.5	0	New lesion
MS5	Female	38	89	IFN-beta sc.	3.5	2.0	1	stable
MS6	Female	47	1	None	1.5	1.5	0	New lesion
MS7	Male	35	16	None	1.0	1.0	0	New lesion
MS8	Male	25	55	Mitoxantrone	2.0	2.5	1	New lesion

The table shows gender, age, the previous treatment, and the EDSS scores at therapy initiation (baseline) and after 12 months. The duration from the diagnosis of definite MS to the start of GA therapy in months (disease duration) is also given. The gender ratio was 6:2 (female: male) and the average age at study onset was 38.9 ± 7.6 years (mean ± SD). Three patients had a relapse during the first 12 months after GA treatment initiation. Five patients showed a new brain lesion in the follow-up compared to pre-treatment. cMRI: cranial magnetic resonance imaging, EDSS: expanded disability status scale, sc.: subcutaneous, SD: standard deviation.

### Monocyte isolation, RNA preparation and gene expression profiling

Monocytes were isolated from the blood samples using erythrocyte lysis buffer (Qiagen, Hilden, Germany), followed by magnetic-activated cell sorting (MACS). The CD14+ cells were magnetically labeled using CD14 MicroBeads and collected as positively selected cell fraction using the autoMACS Separator (Miltenyi Biotec, Teterow, Germany). Total RNA was then isolated from the monocytes using RNeasy columns (Qiagen, Hilden, Germany). RNA concentrations were measured using a NanoDrop 1000 spectrophotometer (Thermo Fisher Scien-tific, Wilmington, DE, USA) and their integrity was assessed with an Agilent 2100 Bioanalyzer using RNA 6000 Pico LabChips. The microarray experiments were performed with an Affymetrix platform. HG-U133 Plus 2.0 GeneChips were used for this analysis. From each sample preparation, total RNA amounts ranging from 100 ng to 200 ng were used as starting material. RNA was converted to cDNA and later into biotinylated cRNA using the MessageAmp II-Biotin Enhanced Kit (Ambion, Foster City, CA, USA). The cRNA molecules were fragmented and 15 μg of cRNA were hybridized onto the GeneChips for 16 hours at 45°C. The GeneChips were later washed and stained in the Affymetrix Fluidics Station 450 and scanned with a GeneChip Scanner 3000 7G system. All these procedures were performed according to the manufacturer protocols (Affymetrix, Santa Clara, CA, USA).

### Microarray data pre-processing

Initial data pre-processing and quality control was done using the Affymetrix GeneChip operating software (GCOS 1.4) and MAS5.0 statistical algorithms (Microarray Analysis Suite 5.0) (Affymetrix, Santa Clara, CA, USA). Since the annotation of genes has changed since the development of the microarrays, there are oligonucleotide probes on the chips, which match to no transcript, and probes, which match to multiple transcripts. Therefore, to exclude such probes from the analysis, we used a custom chip definition file (CDF), which was based on the GeneAnnot database version 1.9 (http://www.xlab.unimo.it/GA_CDF/, CDF version 2.1.0) [54]. Each probe set in the custom CDF matches a single gene. Data normalization was done by a loess fit to the data with span = 0.05 using the R package 'affy’.

### Filtering of differentially expressed genes

To filter differentially expressed genes from the data, we applied two criteria. First, we computed paired *t*-tests comparing for each gene the expression at baseline with the expression at one day, one week, one month and two months. In the second analysis, we evaluated the data with the MAID filtering method, which calculates MA plot-based signal intensity-dependent fold-changes (MAID-scores) for each time point comparison [55]. To filter genes that are significantly up-regulated or down-regulated relative to baseline, we combined the MAID-score outcomes with the paired *t-*test outcomes. Genes with |MAID-score| > 2 and *t*-test *P*-value < 0.05 were filtered as differentially expressed.

### Functional analysis of differentially expressed genes

To investigate the functions of the filtered genes, we performed a Gene Ontology (GO) term enrichment analysis, which was based on the association of functional annotations for each gene in the GO database. GOstats [56], a Bioconductor software package, was the application used to test GO terms for overrepresentation. GOstats computes a probability based on a hypergeometric distribution, which assesses whether the number of filtered genes associated with the term is larger than expected by chance. As a reference, that is, the gene universe, we used all genes that were measured with the HG-U133 Plus 2.0 microarrays (n = 18,862).

### Text mining-based gene interaction network analysis

A gene interaction network was constructed for the filtered genes using the Pathway Studio software version 7.1 from Ariadne Genomics (Rockville, MD, USA) [57]. This software allowed the extraction of gene interactions that were automatically obtained from the literature by text mining. The information about interactions between the genes was exported from the Pathway Studio software as a table, which contains nodes (genes) and edges (interactions) as the building blocks of the gene network. Cytoscape, which is an open source software [58], was used to visualize the network. The edges were represented in various shapes to display the type of the interaction (positive, inhibitory and binding).

## Results

### Patient and sample information

Eight patients were included in this study (six females and two males). The patients were 38.9 ± 7.6 years of age (mean ± standard deviation) and had a mean EDSS of 1.6 (1.0 to 3.5) after a mean disease duration of 26.6 (0 to 89) months (Table 1). All patients started GA treatment at standard dose. During the follow-up period of 12 months, three patients had one relapse each and the other five patients had no relapse. There was only a moderate increase in disability when comparing the EDSS at study onset (baseline) with the EDSS after a follow-up of one year (Table 1). One patient discontinued the therapy in this period of time: MS5 had a severe relapse soon after study onset and, therefore, switched to natalizumab therapy (Tysabri, Biogen Idec, Weston, MA, USA) after three months. Cranial MRI scans were done for seven of the eight patients before the start of GA therapy as well as after a mean follow-up of 17.6 ± 9.5 months. Despite the therapy, five patients each had one new lesion.

From each blood sample, monocytes were isolated by MACS separation. For quality control, we analyzed the mRNA levels of genes, which are known to be specifically expressed by different blood cell types [59]. This revealed high CD14+ monocyte purities (Additional file 1: Figure S1). The RNA that was isolated from the monocytes was in general of high quality with an average RNA integrity number (RIN) of 9.6. The quality of RNA was poor for three samples (MS3/1 month, MS6/1 week, MS7/1 week) and so they were excluded from further analyses.

### Differentially expressed genes

**Figure 1:**
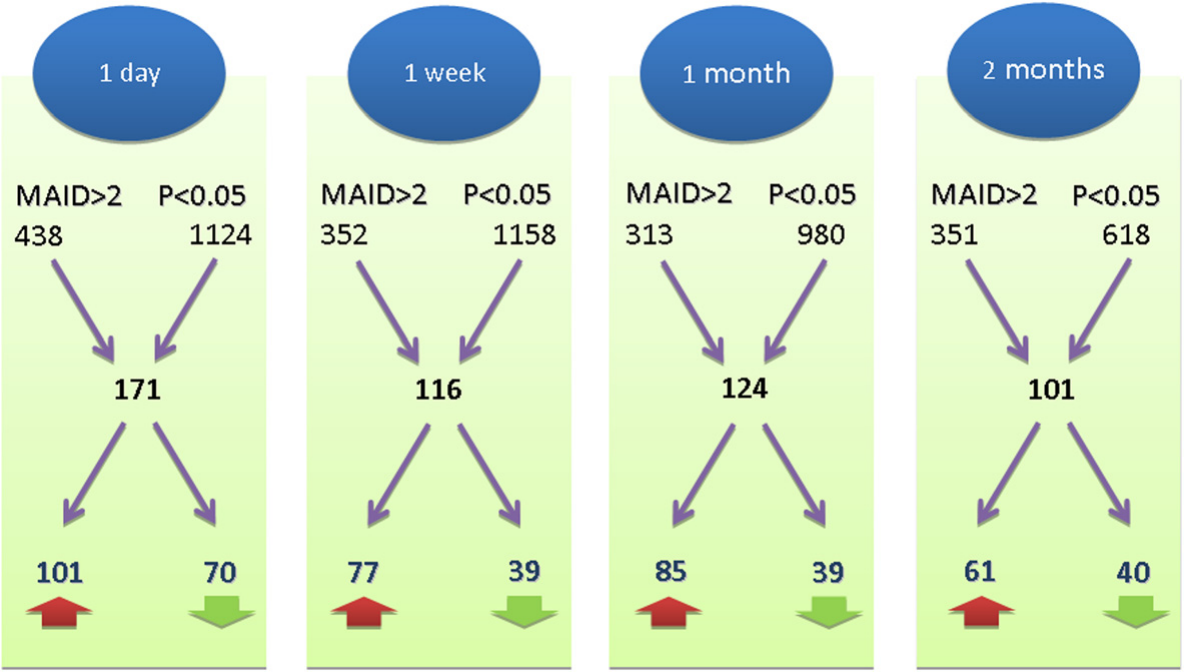
**Longitudinal study design and gene filtering results.** Blood was sampled from eight patients at five different time points: before the start of GA treatment as well as after one day, one week, one month and two months. The expression of 18,862 genes was measured in monocytes with Affymetrix microarrays, and transcript levels during therapy were compared to the pre-treatment levels. *t*-test *P*-values and MAID-scores were used to determine differentially expressed genes. For instance, when comparing the baseline levels with the expression levels after one day, 438 genes survived the MAID analysis criterion (|MAID-score| > 2) and 1,124 genes survived the paired *t*-test criterion (*P*-value < 0.05). In combination, this resulted in 171 filtered genes: 101 were up-regulated (red arrow) and 70 were down-regulated (green arrow). For each time point comparison, a similar number of genes were filtered. However, the overlap of these four gene lists was relatively small (n = 45). When taken together, 463 different genes were identified to be differentially expressed within the first two months of GA therapy.

The pre-processing of the Affymetrix microarray data resulted in transcript levels for 18,862 different genes and 37 different samples. The data are available in the Gene Expression Omnibus (GEO) database with the accession number 'GSE42763’. The filtering of genes differentially expressed in response to GA therapy resulted in a gene list for each time point comparison. In total, 171 genes met the filtering criteria for one day, 116 genes for one week, 124 genes for one month and 101 genes for two months versus baseline (Figure 1, Additional file 2: Table S1). These four gene lists, when aggregated, resulted in 463 different genes (293 up-regulated and 170 down-regulated genes). We observed no accumulation of gene regulatory effects in the course of the therapy since similar numbers of genes were filtered at early (within the first week) and later time points (after one and two months). Moreover, unexpectedly, we found no stable signature of GA-responsive genes since only 45 of the 463 genes were repeatedly identified as differentially expressed, and there was no gene modulated in expression at all time points during therapy.

**Figure 2:**
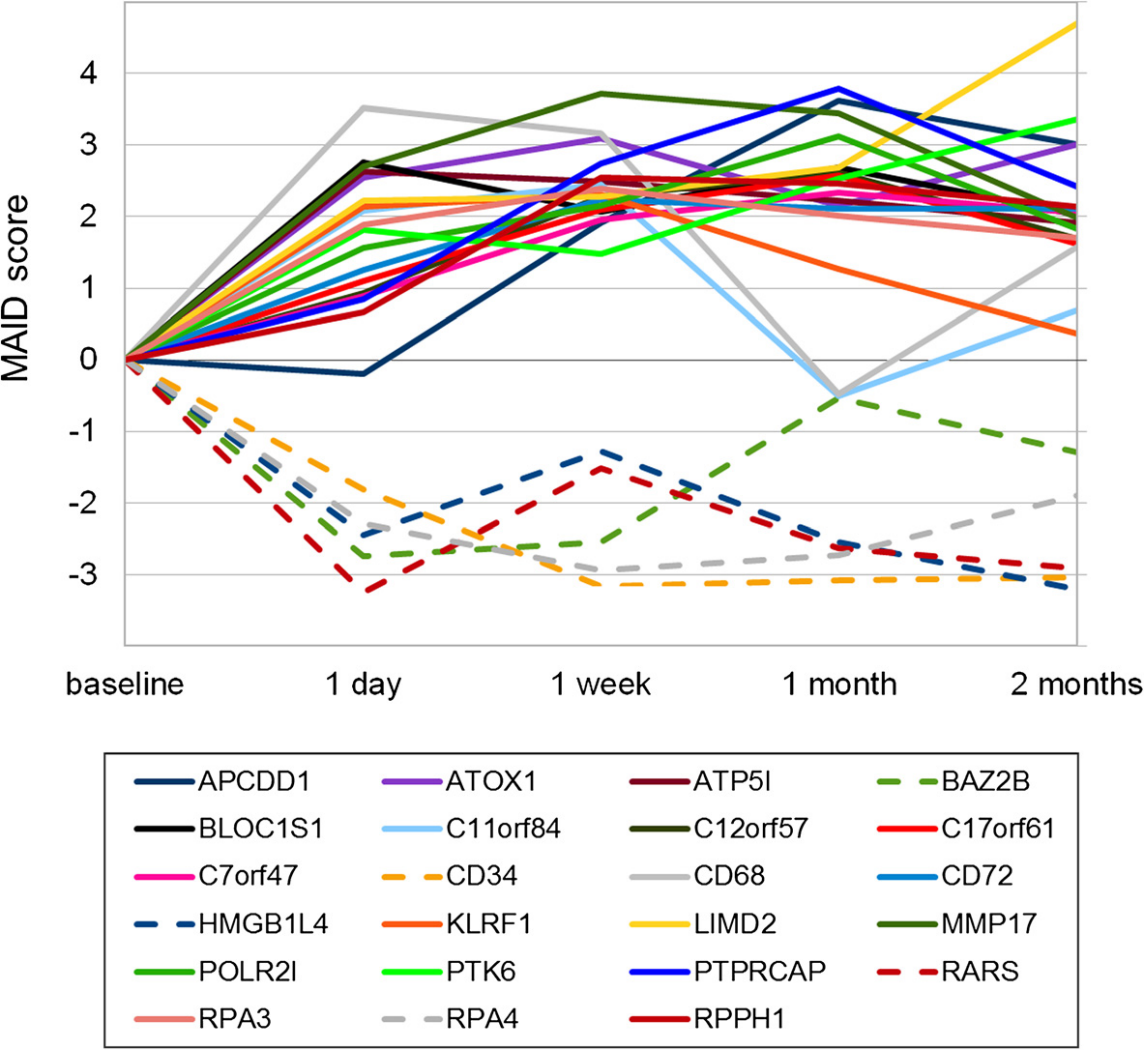
**Dynamics of 23 genes consistently modulated in expression during glatiramer acetate (GA) therapy.** A subset of the 463 filtered genes appeared repeatedly as differentially expressed in consecutive time point comparisons. The graph shows the mean mRNA expression dynamics of 23 genes that were repeatedly found expressed at significantly higher or lower levels across at least two different time points compared to the pre-treatment levels. There were 18 up-regulated genes and 5 down-regulated genes (CD34, RPA4, HMGB1L4, RARS and BAZ2B). The MAID-score, a fold-change variant that is calculated from the data of all patients, is represented on the y-axis and the time points are represented on the x-axis.

To further narrow down the list of genes, we selected only those genes that were expressed at significantly higher or lower levels compared to baseline at two or more consecutive time points. This resulted in a subset of 23 out of the 463 genes. Of these 23 genes, 5 genes were down-regulated (CD34, RPA4, HMGB1L4, BAZ2B and RARS) and 18 genes were up-regulated (for example, ATOX1, BLOC1S1, LIMD2, POLR2I and RPA3). The average mRNA expression dynamics of these genes during GA therapy are shown in Figure 2.

### Functional annotation of the genes

A Gene Ontology (GO) term enrichment analysis was performed to determine the functions, which are characteristic for the genes filtered as differentially expressed in response to GA therapy (n = 463). The GO terms are classified into three major categories: biological process (BP), cellular component (CC) and molecular function (MF). In our analysis, we shortlisted the top 20 overrepresented GO terms according to the *P*-value (Table 2). Several genes appeared under multiple GO terms. The GO terms, which had the most gene members in the list of filtered genes, were 'extracellular region’ (GO:0005576, *P*-value = 2.27E-07) and 'immune system process’ (GO:0002376, *P*-value = 1.19E-04). The GO term 'extracellular region’ contains genes whose protein products are secreted from cells, for example, cell communication molecules, and it was associated to 76 of the 463 genes. Out of them, 45 genes were up-regulated, for example, POMC, MMP17, LTB, XCL1 and APOL3, and 31 genes were down-regulated, for example, CD163, ADAMTS5, TNFSF14, CTSZ and PAM. The GO term 'immune system process’ contained 48 of the genes. Out of them, 35 genes were up-regulated, for example, CD38, CXCL9, CXCL10, IL18 and ICAM2, and 13 genes were down regulated, for example, PTPRC, NCK2, C4BPA, GLMN and ITGAV. There were 22 genes that belonged to both of these GO terms.

**Table 2:** Analysis of gene functions

**Term**	**GO accession**	**ExpCount**	**Count**	**Odds ratio**	***P*-value**
Extracellular region	GO:0005576 (CC)	42	76	2.06	2.27E-07
Cytokine activity	GO:0005125 (MF)	4	15	4.18	1.12E-05
Receptor binding	GO:0005102 (MF)	21	41	2.19	1.85E-05
Extracellular region part	GO:0044421 (CC)	22	42	2.06	5.17E-05
Immune response	GO:0006955 (BP)	17	35	2.19	6.74E-05
Extracellular space	GO:0005615 (CC)	17	34	2.17	9.00E-05
Immune system process	GO:0002376 (BP)	28	48	1.90	1.19E-04
Regulation of lymphocyte activation	GO:0051249 (BP)	5	14	3.30	2.19E-04
Defense response	GO:0006952 (BP)	18	33	2.01	4.38E-04
Regulation of lymphocyte proliferation	GO:0050670 (BP)	2	9	4.27	5.07E-04
Lymphocyte proliferation	GO:0046651 (BP)	3	10	3.89	5.08E-04
T-cell proliferation	GO:0042098 (BP)	2	8	4.75	5.37E-04
Regulation of mononuclear cell proliferation	GO:0032944 (BP)	2	9	4.23	5.44E-04
Cytokine receptor binding	GO:0005126 (MF)	4	12	3.33	5.48E-04
Mononuclear cell proliferation	GO:0032943 (BP)	3	10	3.82	5.76E-04
Regulation of leukocyte activation	GO:0002694 (BP)	5	14	2.97	5.90E-04
Regulation of leukocyte proliferation	GO:0070663 (BP)	2	9	4.14	6.26E-04
Leukocyte proliferation	GO:0070661 (BP)	3	10	3.72	6.92E-04
Regulation of cell activation	GO:0050865 (BP)	5	14	2.82	9.40E-04
Regulation of T-cell proliferation	GO:0042129 (BP)	2	7	4.93	9.56E-04

The top 20 Gene Ontology (GO) terms that were significantly overrepresented for the list of filtered genes (n = 463 genes) are shown in the table sorted by *P*-value. For example, in the second row, 'cytokine activity’ is listed as an overrepresented GO term. 'GO:0005125’ is the corresponding GO database accession. The third and fourth column give the expected number ('ExpCount’, 4 genes) and the actual number ('Count’, 15 genes) of genes in the filtering result that are associated with 'cytokine activity’. This led to an odds ratio of 4.18 and a *P*-value of 1.12E-05. The GO terms are classified into three major groups: biological process (BP), cellular component (CC) and molecular function (MF).

There were several overrepresented GO terms, which are relevant to monocytes, for instance 'mononuclear cell proliferation’ (GO:0032943, *P*-value = 5.76E-04), 'regulation of mononuclear cell proliferation’ (GO:0032944, *P*-value = 5.44E-04) and 'leukocyte proliferation’ (GO:0070661, *P*-value = 6.92E-04). These GO terms form a hierarchy where a GO term is part of a broader GO term, hence these GO terms share most of the genes. Genes found modulated in expression during GA treatment and belonging to all of these three terms are, for example, CD38, GLMN, IGHM, IL18, NCK2 and PTPRC. Other notable overrepresented GO terms were 'regulation of lymphocyte activation’ and 'cytokine activity’.

### Gene interaction network

**Figure 3:**
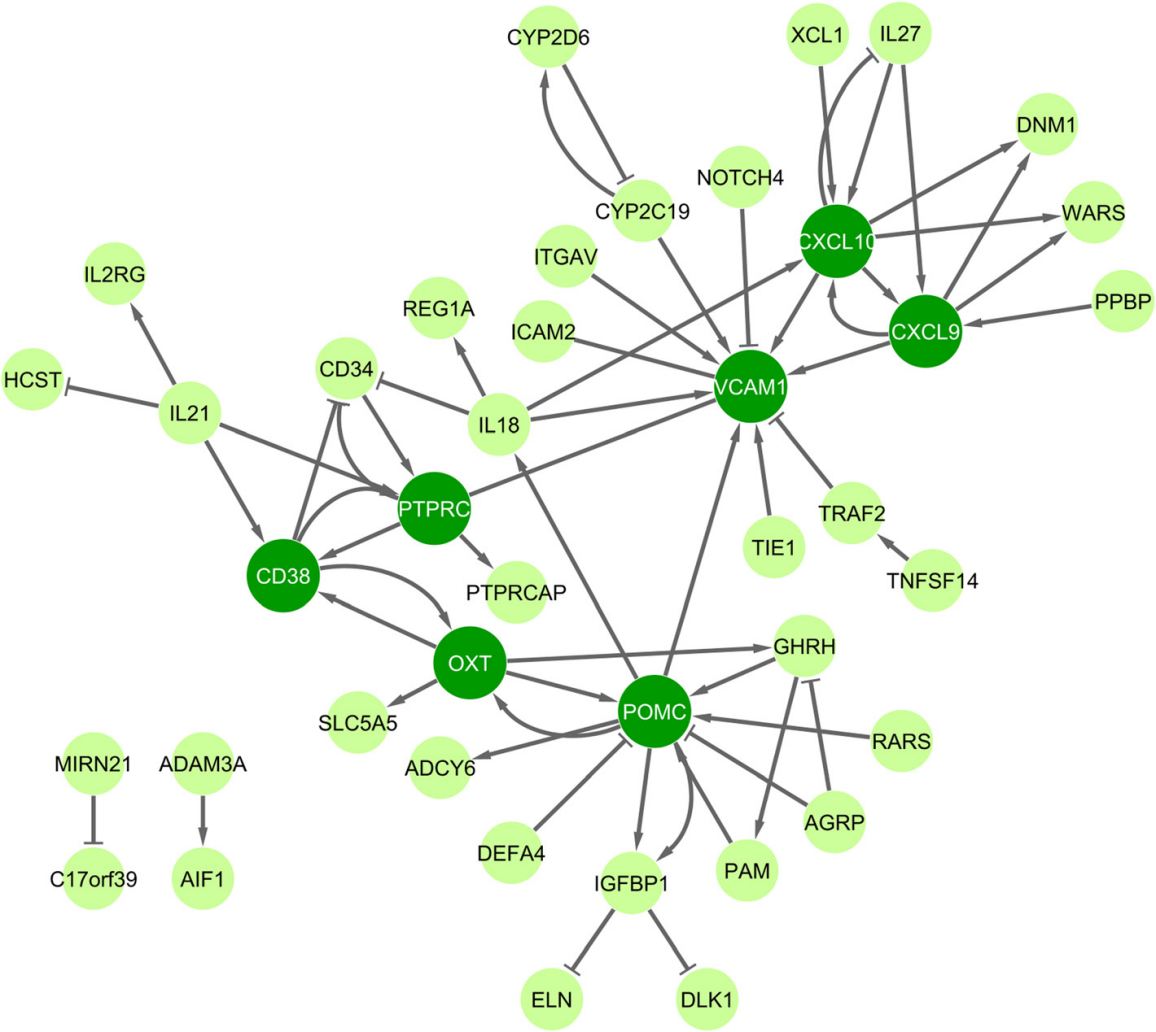
**Interaction network of genes differentially expressed in response to glatiramer acetate (GA).** A gene interaction network was retrieved for the 463 filtered genes using the Pathway Studio software. The software delivered a network of 41 genes (= nodes) with 59 interactions (= edges) between them. The other genes with no interaction are not shown in the figure. Edges are represented in different ways in the network. An arrow (→) means positive effect (n = 43), a 'T’ (┤) means negative effect (inhibition, n = 14) and a line (─) means binding (n = 2). The seven nodes, which are highlighted in dark green, are involved in the regulation of many other genes and thus may play an important role in the molecular mechanisms of action of GA. The network is available as a Cytoscape session file from the authors upon request.

The unified list of 463 genes was used as input for the Pathway Studio software to gather pair-wise interactions between them. The output resulted in 41 genes (= nodes) with 59 interactions (= edges). The interactions were visualized as a network (Figure 3). The edges vary according to their interaction type. There were 43 positive regulatory interactions, 14 inhibitory interactions and 2 binding interactions. The network revealed different interaction clusters. Seven of the genes, CXCL10, CXCL9, VCAM1, POMC, OXT, PTPRC and CD38, possess the majority of the interactions with the other genes in the network: except PTPRC, they all appeared as up-regulated during GA therapy.

POMC, a polypeptide hormone precursor, had 12 interactions and is therefore the most connected gene in the network. For instance, VCAM1, OXT, IL18 and ADCY6 (which were up-regulated) and IGFBP1 (which was down-regulated) are influenced by POMC according to the literature-based interaction network. The second cluster is based on VCAM1, a vascular cell adhesion protein, which had 11 interactions. It is regulated by IL18, POMC, ITGAV, CYP2C19, CXCL10, CXCL9, TRAF2, NOTCH4 and TIE1. Of these, ITGAV was down-regulated and all the other genes were up-regulated in response to GA treatment. The third cluster is formed by OXT, which regulates CD38, SLA5A5, GHRH and POMC. The fourth cluster is based on two up-regulated chemokines, CXCL9 and CXCL10, which together had 14 interactions. They have a feedback loop between them, and CXCL10 is further regulated by IL18, IL27 and XCL1, which were all up-regulated after the first week of GA therapy. The network also shows that PTPRC (= CD45) is linked with PTPRCAP, CD34, CD38, IL21 and VCAM1. CD38 had six interactions: CD38 inhibits CD34, is regulated by IL21, and has feedback loops with OXT and PTPRC (Figure 3).

## Discussion

This study focused on the *in vivo* effects of GA therapy on the gene expression of monocytes, which are the precursors of macrophages and dendritic cells. The monocytes were obtained from the blood of RRMS patients and we compared the transcript levels before and after the start of GA therapy. For each patient, the gene expression was measured at five time points up to two months into therapy. In these data, we identified 463 genes as up-regulated or down-regulated during therapy compared to pre-treatment levels. More than a hundred genes were filtered at early (after one day) and later (after two months) time points post treatment initiation. However, relatively few genes were repeatedly found to be differentially expressed in the course of time. This indicated that the gene regulatory effects of GA on monocytes are rather modest and no stable gene expression signature could be seen. Nevertheless, the mRNA changes of some genes might tell us something about GA’s molecular mechanisms of action.

Compared to our study, in the gene expression study by Achiron *et al.*, only two time points were compared: before and after three months of GA treatment [52]. Therefore, the variability in the mRNA dynamics early during therapy possibly has been underestimated so far. Moreover, Achiron *et al.* studied the gene expression changes in PBMC of RRMS patients, whereas we studied monocytes. Using Affymetrix microarrays, they found 480 genes to be differentially expressed in response to GA administration, with the main effects being related to antigen-activated apoptosis, inflammation, adhesion, and MHC class I antigen presentation [52]. As in our study, there were more up-regulated than down-regulated genes. However, when comparing their gene list (n = 480) with ours (n = 463), only five genes (BAT1, ELOVL5, ETV7, MT1E and PCBD1) were in common. One explanation for that might be that GA possibly acts primarily on other subsets of circulating cells, for example, by altering the functional properties of (autoreactive) T-cells. Therefore, different gene regulatory effects might be seen in PBMC than in monocytes from GA-treated patients.

On the other hand, a recent cross-sectional study by Ottoboni *et al.* found no significant differences in the PBMC RNA profiles of untreated and GA-treated patients [60]. In their study, they took into account the multiple testing by computing false discovery rates (FDR) [61]. In our data set, if we set the threshold for statistical significance at FDR < 0.05, also no gene remained as differentially expressed during GA therapy. Instead, we chose less conservative filtering criteria to detect even moderate shifts in the gene expression of monocytes, but, in consequence, also some weakly modulated and less expressed genes survived the filtering for GA-responsive genes (Additional file 2: Table S1). A subset of 23 genes was repeatedly identified to be up-regulated or down-regulated at different time points during therapy (Figure 2). These 23 genes might represent good candidates of molecular markers of GA activity. However, for confirmation, a larger independent study with more sensitive measurement techniques such as real-time PCR is needed. As another limitation of our study, we did not measure the transcript levels in the long-term after the first two months of treatment. Possibly, the full modulation of immunological processes by GA may require more time. It was also beyond the scope of the present study to examine whether the individual gene expression profiles are associated with the clinical data (for example, relapse rate, EDSS and MRI).

We performed a GO term enrichment analysis for the list of 463 filtered genes to classify them according to their functions and the biological processes they are involved in (Table 2). Overrepresented GO terms included 'lymphocyte proliferation’ and 'regulation of T-cell proliferation’. These findings are consistent with earlier studies that showed that GA suppresses lymphocyte proliferation through modulation of monocytes and monocyte-derived dendritic cells, thereby reducing the number of autoreactive T-cells [38,62,63]. Members of these GO terms are, for example, cytokines such as IL18 and TNFSF14. Additionally, we searched for interactions between the genes and retrieved 59 interactions. Of note, these interactions were obtained by literature mining. Therefore, the gene network (Figure 3) shows direct as well as indirect regulatory effects on the transcript and protein level. Seven genes had several interactions (CXCL9, CXCL10, VCAM1, POMC, OXT, PTPRC and CD38).

The network contained different cytokines, for example, CXCL9 and CXCL10 whose expression was increased one day after the start of GA therapy. These two chemokines bind to the CXCR3 receptor and are involved in the recruitment of immune cells to sites of inflammation, principally acting on activated CD4+ Th1 cells, CD8+ T-cells and natural killer (NK) cells [64,65]. A previous study already showed that the transcription of CXCL10 is induced in PBMC after GA administration [66]. Both CXCL9 and CXCL10 were also described to be modulated in expression in blood during treatment with IFN-β [67]. Therefore, both drugs seem to affect the chemokine gradient between brain lesions and the peripheral immune compartment. The cytokine group further included interleukins (IL18, IL21, IL25 and IL27), which were all found to be up-regulated in our data set. Of those, IL18 and IL27 play important roles in the differentiation and expansion of naive CD4+ T-cells [68,69]. Moreover, TNFSF14, a member of the TNF cytokine family, was down-regulated one day after the first GA injection. TNFSF14 is known to function as a costimulatory factor regulating the activation of T-cells [70]. A single nucleotide polymorphism within an intron of the TNFSF14 gene is associated with MS susceptibility [71,72]. Another related member of the TNF family, LTB (lymphotoxin-β), was also filtered as differentially expressed. Both LTB and TNFSF14 bind to the LTBR receptor, and they provide communication links in innate and adaptive immune responses [73,74]. However, the transcriptional modulation of these cytokines was not stable over time and it thus remains unclear how these immunoregulatory effects may exactly contribute to the mechanisms of action of GA.

The interaction network of filtered genes also contains several cell adhesion receptors, for example, ITGAV, ICAM2 and VCAM1. ITGAV encodes the integrin αV, an integral membrane protein that can interact with a variety of extracellular matrix ligands. Integrins orchestrate monocyte differentiation into macrophages, and they play a role in macrophage adhesion, migration and tissue infiltration [75]. Moreover, ITGAV is known to mediate proinflammatory cytokine synthesis in human monocytes [76]. It was expressed at lower levels after the first injection of GA, which may reflect the previously described shift in the gene expression of monocytes towards an anti-inflammatory profile [34,39].

Other genes in the network are implicated in quite different biological processes, for example, RARS, WARS, PTPRC, PTPRCAP and MIRN21. RARS and WARS encode the arginyl- and tryptophanyl-tRNA synthetase, respectively. They catalyze the amino acid attachment to cognate tRNAs during protein synthesis. However, besides their role in protein translation, biologically active fragments of WARS were also discovered to be involved in angiogenesis signaling pathways [77]. PTPRC is a transmembrane glycoprotein associated with PTPRCAP. Both genes were found modulated in expression after one month of treatment compared to baseline. PTPRC functions as a regulator of cytokine receptor signaling and influences cellular processes such as cell proliferation [78]. Upon activation of monocytes, proteolytic processing of PTPRC results in a protein fragment, which is released and acts as an inhibitor of T-cell proliferation [79]. The microRNA gene MIRN21 was expressed at higher levels after two months compared to pre-treatment levels. MicroRNAs are involved in the post-transcriptional regulation of gene expression. The transcript MIRN21 harbors the mature microRNA hsa-miR-21, which has been shown to be up-regulated in active MS lesions [80] and to be higher expressed in PBMC of RRMS patients versus controls [81]. MicroRNAs in MS and therapy are worthy of being explored in more detail [82,83]. So far, there is only one study that has specifically investigated whether GA therapy affects the levels of mature microRNAs, but this study was limited to five selected microRNAs [84].

Other studies demonstrated that GA treatment leads to a change in the properties of monocytes from pro-inflammatory type I monocytes to anti-inflammatory type II monocytes [34]. However, although some cytokines were differentially expressed during GA therapy, the mRNA levels of TNF-α, TGF-β, IL10, IL12, IL1-β and IL1RA were not affected in our data set. Therefore, we could not observe a clear cytokine shift in monocytes in response to GA. One reason for that might be that in our study monocytes were isolated from peripheral blood samples of MS patients, whereas, in contrast, in the study by Weber *et al.* the monocytes were separated from the spleen of mice with EAE [34]. Moreover, our study was restricted to mRNA transcripts and we did not measure the amounts of the encoded proteins and their splice variants, whereas other groups analyzed the protein levels of monocytes in culture after *in vitro* stimulation [35,36,38]. Burger *et al.* studied the effects of GA on the transcription of two genes (IL1-β and IL1RA) in monocytes [39,40]. However, their results might not be reflected in our data since they used monocytes from blood donors and stimulated these cells *in vitro* with GA. In our study, we could neither identify a stable signature of differentially expressed genes nor a solid evidence of an increase of type II monocytes within the first two months of therapy. This finding cannot be explained by just the relatively small number of recruited patients. Therefore, we conclude that the *in vivo* effects of GA on monocytes in the peripheral blood are rather modest and variable. It is likely that most of the effects occur at the injection sites or in the draining lymph nodes where (MBP-specific) T-cells as well as monocytes and professional APC are confronted with GA peptides. Additionally, since GA is a mixture of randomly synthesized peptides, the molecular effects might be somewhat different from injection to injection. All this makes it quite a challenge to study the drug’s molecular mechanisms of action. Further studies are needed to better understand how GA modulates the immune system, also because new drugs similar to GA are currently tested for RRMS (for example, ATX-MS-1467, Apitope Technology Ltd., Bristol, UK).

## Conclusions

There is a lack of transcriptome studies on the effects of GA in MS patients. Here, we presented the first genome-wide and cell type-specific analysis of the mRNA dynamics during GA therapy. Using microarrays, we longitudinally measured the gene expression of monocytes for a small patient group at five different time points. We identified 463 genes as differentially expressed within the first two months of GA treatment, the majority being associated with immunological processes (for example, cytokines). However, the changes in gene expression were not sustained over time, and most genes were seen up-regulated or down-regulated only once. Therefore, GA seems to have only little gene regulatory effects on monocytes. Our study nevertheless delivered some genes that are worth investigating in future studies regarding the molecular mechanisms of GA therapy in the peripheral blood of MS patients.

## Abbreviations

APC: Antigen-presenting cell; APL: Altered peptide ligand; BP: Biological process; CC: Cellular component; CDF: Chip definition file; CNS: Central nervous system; DGN: German society of neurology; EAE: Experimental autoimmune encephalomyelitis; EDSS: Expanded disability status scale; EDTA: Ethylenediaminetetraacetic acid; FDR: False discovery rate; GA: Glatiramer acetate; GEO: Gene expression omnibus; GO: Gene ontology; HSP: Heat shock protein; IFN: Interferon; IL: Interleukin; MACS: Magnetic-activated cell sorting; MAID: MA plot-based signal intensity-dependent fold-change criterion; MBP: Myelin basic protein; MF: Molecular function; MHC: Major histocompatibility complex; MRI: Magnetic resonance imaging; MS: Multiple sclerosis; NK: Natural killer; PBMC: Peripheral blood mononuclear cells; RIN: RNA integrity number; RRMS: Relapsing-remitting multiple sclerosis; Sc: Subcutaneous; SD: Standard deviation; TCR: T-cell receptor; Th: T helper cell; Treg: T regulatory cell.

## Competing interests

UKZ received research support as well as speaking fees from Teva, Biogen Idec, Bayer HealthCare, Novartis, Almirall, Merck Serono and Sanofi-Aventis. MH received speaking fees from Bayer HealthCare, Teva and Novartis. Meanwhile, RHG is a salaried employee of Teva, and he was a salaried employee of Novartis. MT, BKP, IS, DK and H-JT declare no potential conflict of interest.

## Authors’ contributions

UKZ and H-JT inspired and directed the work. IS was responsible for clinical documentation. The lab experiments were performed by RHG, DK, BKP and MT. MH and RHG carried out the analysis and interpretation of the data. MT and MH drafted the paper and prepared all tables and figures. H-JT and UKZ contributed to the writing of the manuscript. All authors read and approved the final manuscript.

## Supplementary Material

Additional file 1: Figure S1 Analysis of the purity of the isolated monocytes. (A) We visualized the measured transcript levels of five selected genes, which are specifically expressed in different blood cell types, namely CD14 (monocytes), CD3D (T-cells), MS4A1 (for example, B-cells), KLRD1 (for example, NK cells) and HBD (erythrocytes). CD14 was expressed at very high levels (> 18,000) in all 37 samples of the microarray data set, whereas the other genes were expressed at very low levels (< 400). This demonstrates high purity of the monocytes isolated by MACS. (B) We used the Affymetrix microarray data by Novershtern *et al.*[59] to compare the mRNA levels of these genes in distinct human hematopoietic cell populations, for example, CD4+ and CD8+ T-cells, B-cells and monocytes. The preprocessed data were downloaded from the GEO database (accession number 'GSE24759’). The bar charts show the mean ± standard error of the expression values of the respective probe sets (given in brackets) in 14 different cell types. A limited purity of the isolated monocytes would be noticeable in figure A, because CD3D, MS4A1, KLRD1 and HBD are highly expressed in other cells of the blood. Click here for file

Additional file 2: Table S1 Filtered differentially expressed genes. This Excel file contains four gene lists, which provide the genes that were identified as differentially expressed after one day (t1), one week (t2), one month (t3) or two months (t4) of GA therapy when compared to baseline levels (t0). For each gene, identifiers for the databases GeneCards, Entrez and HGNC (gene symbols) are provided together with their official full names. Additionally, the mean gene expression levels (averaged over the patients) and the respective standard deviations (SD) are given for all compared time points as well as the computed *t*-test *P*-values and MAID-scores. The column 'Regulation’ denotes, if the gene was found up-regulated or down-regulated in response to GA relative to baseline. Click here for file
