# Supplementary figures and images for: Glatiramer acetate treatment effects on gene expression in monocytes of multiple sclerosis patients
Source: J Neuroinflammation. 2013 Oct 17;10:126. doi: 10.1186/1742-2094-10-126 (PMC3852967; doi:10.1186/1742-2094-10-126)

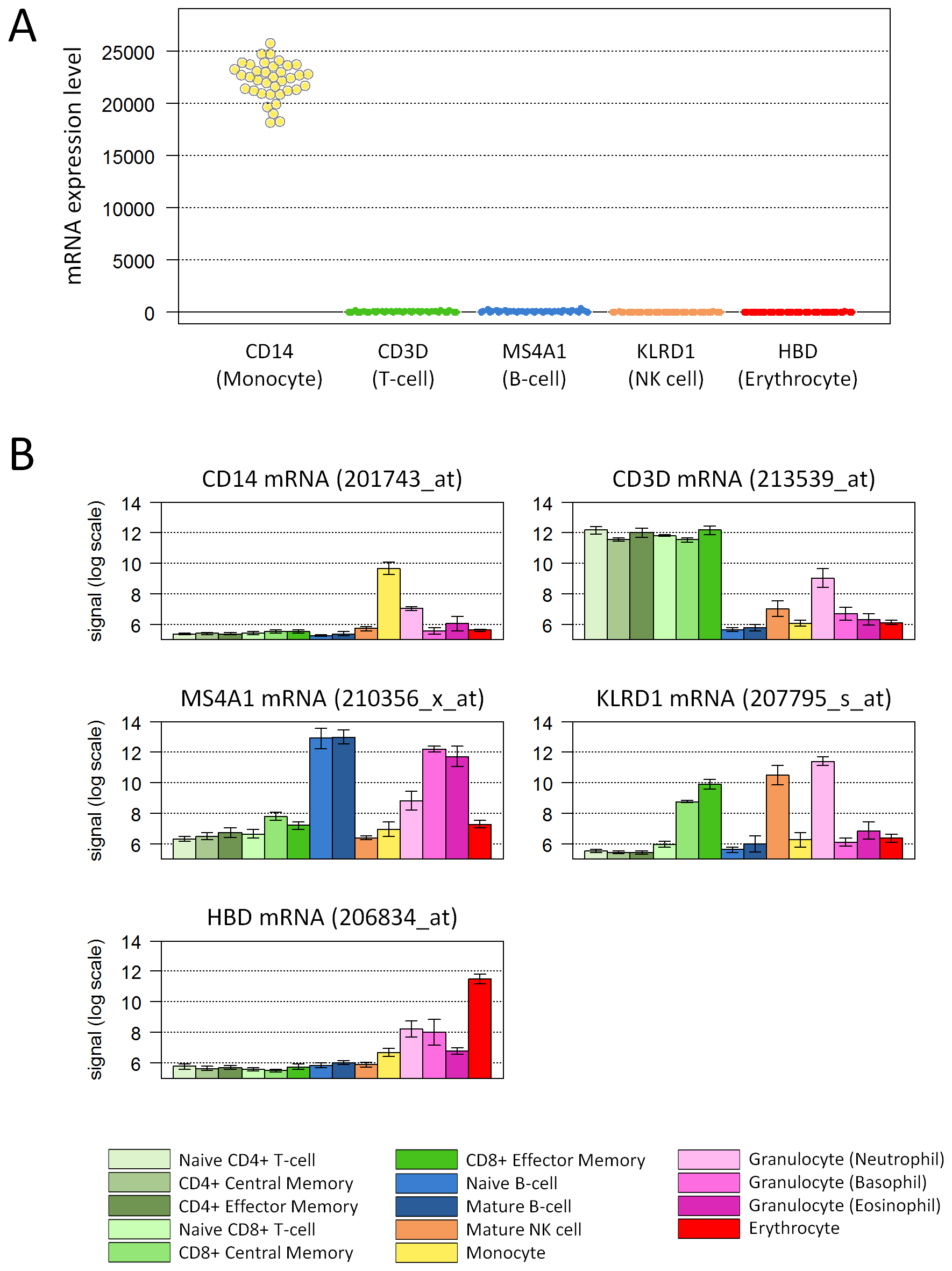

Supplement: Additional file 1: Figure S1 — Analysis of the purity of the isolated monocytes. (A) We visualized the measured transcript levels of five selected genes, which are specifically expressed in different blood cell types, namely CD14 (monocytes), CD3D (T-cells), MS4A1 (for example, B-cells), KLRD1 (for example, NK cells) and HBD (erythrocytes). CD14 was expressed at very high levels (> 18,000) in all 37 samples of the microarray data set, whereas the other genes were expressed at very low levels (< 400). This demonstrates high purity of the monocytes isolated by MACS. (B) We used the Affymetrix microarray data by Novershtern et al.[59] to compare the mRNA levels of these genes in distinct human hematopoietic cell populations, for example, CD4+ and CD8+ T-cells, B-cells and monocytes. The preprocessed data were downloaded from the GEO database (accession number 'GSE24759’). The bar charts show the mean ± standard error of the expression values of the respective probe sets (given in brackets) in 14 different cell types. A limited purity of the isolated monocytes would be noticeable in figure A, because CD3D, MS4A1, KLRD1 and HBD are highly expressed in other cells of the blood. [file 1742-2094-10-126-S1.tiff]
